# Implementation of pharmacokinetic and pharmacodynamic strategies in early research phases of drug discovery and development at Novartis Institute of Biomedical Research

**DOI:** 10.3389/fphar.2014.00174

**Published:** 2014-07-28

**Authors:** Tove Tuntland, Brian Ethell, Takatoshi Kosaka, Francesca Blasco, Richard Xu Zang, Monish Jain, Ty Gould, Keith Hoffmaster

**Affiliations:** ^1^Metabolism and Pharmacokinetics, Genomics Institute of Novartis Research FoundationSan Diego, CA, USA; ^2^Metabolism and Pharmacokinetics, Novartis Institute of Biomedical ResearchHorsham, West Sussex, UK; ^3^Metabolism and Pharmacokinetics, Novartis Institute of Tropical DiseasesSingapore, Singapore; ^4^Metabolism and Pharmacokinetics, Novartis Institute of Biomedical ResearchEmeryville, CA, USA; ^5^Metabolism and Pharmacokinetics, Novartis Institute of Biomedical ResearchCambridge, MA, USA

**Keywords:** pharmacodynamics, PK-PD modeling, drug discovery, DMPK, Novartis, pharmacokinetics

## Abstract

Characterizing the relationship between the pharmacokinetics (PK, concentration vs. time) and pharmacodynamics (PD, effect vs. time) is an important tool in the discovery and development of new drugs in the pharmaceutical industry. The purpose of this publication is to serve as a guide for drug discovery scientists toward optimal design and conduct of PK/PD studies in the research phase. This review is a result of the collaborative efforts of DMPK scientists from various Metabolism and Pharmacokinetic (MAP) departments of the global organization Novartis Institute of Biomedical Research (NIBR). We recommend that PK/PD strategies be implemented in early research phases of drug discovery projects to enable successful transition to drug development. Effective PK/PD study design, analysis, and interpretation can help scientists elucidate the relationship between PK and PD, understand the mechanism of drug action, and identify PK properties for further improvement and optimal compound design. Additionally, PK/PD modeling can help increase the translation of *in vitro* compound potency to the *in vivo* setting, reduce the number of *in vivo* animal studies, and improve translation of findings from preclinical species into the clinical setting. This review focuses on three important elements of successful PK/PD studies, namely partnership among key scientists involved in the study execution; parameters that influence study designs; and data analysis and interpretation. Specific examples and case studies are highlighted to help demonstrate key points for consideration. The intent is to provide a broad PK/PD foundation for colleagues in the pharmaceutical industry and serve as a tool to promote appropriate discussions on early research project teams with key scientists involved in PK/PD studies.

## Introduction

Effective and successful pharmacokinetics/pharmacodynamics (PK/PD) studies during drug discovery and development phases require input from scientific experts in complementary disciplines in the pharmaceutical industry. In the majority of cases, the pharmacodynamic portion of PK/PD studies (e.g., animal dosing and measurement of response) are conducted by pharmacology laboratories within a given disease area whereas the measurement of concentrations and evaluation of pharmacokinetics are conducted by DMPK laboratories. In some cases pharmacokinetics are not determined in the same animals used in the PD study. Rather, the PK and PD datasets might be generated completely independent of each other, not only in different laboratories but also different timeframes. In the latter scenario, generation and reporting of data can happen in isolation, and project teams are then faced with downstream integration and evaluation of results that lack an integrated analysis defining a concentration and effect relationship. Optimally, when PK/PD studies are designed and conducted, the PK/PD analysis, conclusions and interpretations are performed by both DMPK and pharmacology experts, with input from other relevant partners (e.g., formulation and mathematical modeling experts). The resulting report thus reflects integration of all relevant data and addresses the hypothesis or question asked at the outset of the study. The report will capture any assumptions made in the analysis and suggest what subsequent studies the results enable, and reflects shared ownership and responsibility of both the DMPK and pharmacology experts. The major objective of early drug development is to select promising compounds and to identify potentially safe and effective doses and dosing regimens. Integration of PK/PD in early development helps with compound selection and guides creation of an efficient clinical development strategy (Miller et al., 2005).

## Initiate and refine a PK/PD model

PK/PD modeling is a valuable approach to integrate quantitative information about the pharmacologic properties of a compound with its pharmacokinetics. Rational study design is based on the assumption of a causal relationship between exposure to a medication and its therapeutic activity. Such relationships are generally complex. Therefore it is important to design robust preclinical studies that will provide information to build mechanistically relevant PK/PD mathematical models. As data becomes available, initial models can be refined through an iterative process. The ultimate output is a powerful predictive tool based on an in-depth understanding of the requirements for efficacy. A well designed PK/PD study offers a rational approach to efficient and informative drug development and can help the project team to understand the mechanism of action of a drug and select the optimal compound. Applying PK/PD modeling in early discovery and development programs can minimize animal usage, shorten the development time, estimate the therapeutic index, and predict the dose ranges in early clinical testing. PK/PD models allow integration of data from different studies in a logical manner based on the understanding of drug and disease. Drug discovery and development can be viewed as a model building exercise during in which the knowledge of new compounds is continuously updated and used to inform decision-making and drug development strategy (Lalonde et al., 2007).

### Establish effective partnerships of pharmacology and DMPK

A core drug discovery team in the pharmaceutical industry (e.g., disease area pharmacologist, DMPK scientist, biologist and chemist) will often gather information, literature and reports about the current animal experimental model and study designs used in the project. It is essential that a partnership between pharmacologists and pharmacokineticists starts as early as possible in the course of a discovery program, and that the collaboration continues through to the transition of the program to early stage development and beyond into the clinic. It is highly recommended that the team set up an infrastructure for data sharing. Historical data highlighting examples of both success and failure with disease models are valuable additions to this collection, and teams are encouraged to determine whether a mechanistic or disease animal model is suitable for the project. Discussions with partners in pharmacology and on the core team will enable the team to address key questions and aspects of the PK/PD study. Such discussions may include selection of the pharmacodynamic readouts such as biomarker or efficacious endpoints; the study design with respect to dose regimen, time points and PK analysis; and evaluation of the technical limitations and challenges associated with the PK/PD studies. An important consideration is whether robust and clinically validated biomarkers are available. If not (as in case of working with a novel target or rare disease), it may be necessary to evaluate the translatability of preclinical PD biomarker data to the clinical setting. It is equally important to determine whether the PD markers are amenable to quantitative PK/PD, including simultaneous and continuous PK/PD sampling.

### Conduct preliminary PK/PD analysis

One approach to help establish confidence in, and optimize subsequent PK/PD experiments, is to start with a tool or reference compound for which internal or external reports are available. In cases where those data are not available, it may be advantageous to invest adequate resources to generate a complete data package with a reference compound before starting to test a series of novel compounds in the model. Although project teams may see this as a significant investment at a very early stage of the program, extensive early understanding of the relationship between PK and PD will likely decrease the resource investment in the long term. One risk of moving directly into assessment of novel compounds with limited insight on optimal study design is that considerable effort and resources might be spent on a model that is not fully understood, characterized, or optimized based upon the intrinsic pharmacokinetic and pharmacologic properties of the compounds of interest.

The initial goal at this very early stage is to establish fundamental PK/PD principles and hypotheses. Care must be taken to analyze the data and draw first conclusions and establish a working hypothesis that can be tested by subsequent study design. Ultimately, the goal with studies using a reference or tool compound is to understand the driving force(s) for response, i.e., the relationships between drug concentration and PD readouts. It is advisable to set up a PK/PD model using the most relevant matrix (e.g., blood/plasma or target tissue) which would yield a clearly defined dose-response relationship. Once a PK/PD model has been validated with a suitable tool compound, the team needs to establish if data from the reference compound can be extrapolated to future molecules.

### Define a PK/PD hypothesis

Once a preliminary PK/PD analysis has been completed, translation of this understanding into a sound scientific hypothesis and PK/PD strategy for the project will follow. The analysis will allow the scientists to determine if meaningful interpretations and decisions can be made on the basis of the PK/PD studies. This can be achieved in a process where contributing scientists meet repeatedly to discuss data and evaluate whether further optimization is possible or necessary. Validation of PK/PD models using known model compounds may be required to ensure desired outcome and sensitivity. If the PK/PD strategy is found suitable to triage new compounds, integration of the PK/PD analysis into the project workflow is highly encouraged.

### Refine and implement the PK/PD strategy

Early engagement and discussion within the team helps to define an appropriate hypothesis that can be refined as data emerges from preliminary studies. At this stage the team members need to have effective mechanisms in place to exchange and share data. As a drug discovery program matures and more PK/PD data become available, it is beneficial to compile all the data and integrate results from multiple compounds. Relevant data (e.g., *in vitro* EC_50_, *in vivo* exposure, *in vivo* efficacy) from preliminary studies form the basis for selection of compounds that will be profiled in more robust and detailed follow-up PK/PD studies. This in-depth analysis can permit refinement of initial PK/PD hypotheses as well as promote sophisticated modeling with more data-rich datasets. Overall, the key to successful PK/PD studies is the active partnership between the relevant scientists on the project team. The ultimate PK/PD strategy will reflect not only a discussion of the questions outlined above, but also those that emerge from collaborative discussions between DMPK, pharmacology, and the remainder of the project team. The larger team will arrive at a consensus for the role of the PK/PD data to address key scientific questions that is limiting the progression of the program into further development. At this time it is advantageous to initiate plans for translation of PK/PD into the development phase of research.

## PK/PD study design

**Figure 1:**
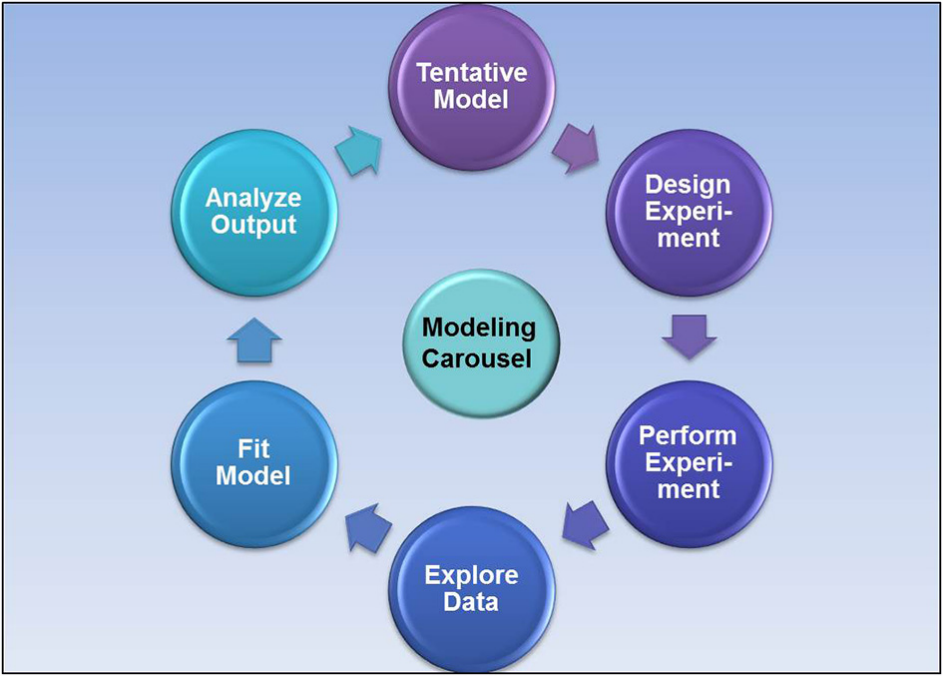
**The iterative process of PK/PD modeling in drug discovery**.

The typical steps involved in the design of PK/PD studies are as follows: First, *in vitro* pharmacological and *in vivo* pharmacokinetic data are collected to help design a PK/PD study protocol. An acute pilot PK/PD model is then conducted to examine the exposure-response relationship. The acute disease models are fairly simple in scope and of short duration (e.g., single dose, one dose level, sparse sampling, and monitoring a single biomarker) with the objective to select compounds that demonstrate acute efficacy. The set-up and screening with a PK/PD model in drug discovery is typically an iterative process that requires ongoing refinement as new information become available and the project moves forward (Figure 1).

**Figure 2:**
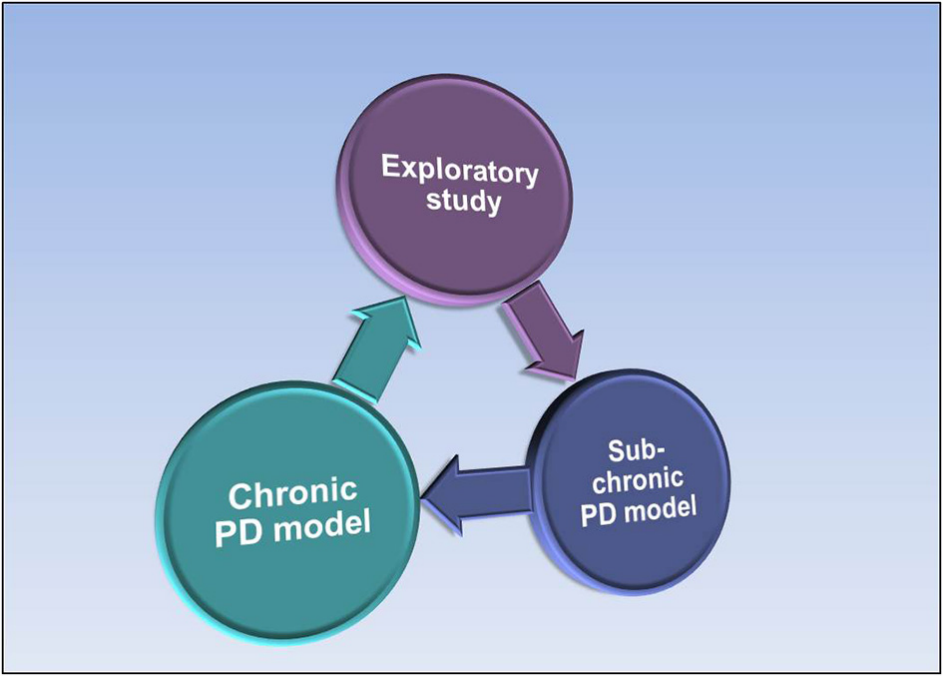
**Progression from acute exploratory PK/PD studies to subchronic and chronic PK/PD studies as drug candidates are identified and profiled**.

PK/PD models are continuously updated throughout different stages of drug development to incorporate relevant new data (Rajman, 2008). Once suitable drug candidates are identified, sub-chronic main PK/PD studies are performed to establish dose-exposure-response relationships and the effective plasma target concentration ranges. Sub-chronic disease models involving repeated dosing for days at multiple dose levels may be utilized to determine the effective concentration range of the compounds. Finally, full chronic disease models are conducted on promising drug candidates to determine the minimum efficacious dose and the relationship between steady-state exposure levels and sustained efficacy (Gabrielsson et al., 2009). Chronic disease models, often complex in nature and of long duration (e.g., 2 weeks daily dosing at multiple dose levels, frequent sampling in blood and target tissues, monitoring of multiple biomarkers) will follow at a later stage to fully characterize the exposure–response relationship (Figure 2). The outcome of the mechanistic biomarker and disease models serve as feedback or validation of the selection process of compounds in earlier screens such as different *in vitro* assays.

Prior to starting a PK/PD study, it is imperative to define the objectives of the study and identify strengths, weaknesses, and gaps in results that might be obtained from the study. It is advisable for teams to consider the correlation of *in vitro* data and *in vivo* efficacy and to understand the PK in the animal test species. Furthermore, it is important to select a relevant, sensitive and reproducible PD read-out, and to appreciate effects of time on the PD read-out. The approaches may differ depending on the stage of the program, previous understanding of the concentration-effect relationships, and the data available a priori to guide the study design. Careful planning of the study design with input from DMPK, pharmacology, and relevant team members will be beneficial. The scientists need to agree on the protocol details including route of administration, study duration and sampling frequency. At such time, existing PK and efficacy data may be used to guide the experimental design, which will depend on the stage of the project and the objective of the study. Early stage projects focusing on the discovery of efficacious compounds may apply different PK/PD study methods compared to those used in projects at lead selection or candidate nomination stages. Furthermore, specific design strategies should be implemented in early proof of concept studies of tool compounds, acute disease PK/PD screens to triage discovery compounds, sub-chronic efficacy models, or later stage chronic disease model to thoroughly characterize the exposure-response relationship.

Historically, PK/PD modeling has been applied in the development of small molecule drugs; however, modeling has more recently been successfully applied to characterize the efficacy and disposition of biotherapeutical drugs such as monoclonal antibodies or antibody-drug conjugates (Agoram et al., 2007; Yu et al., 2009; Jumbe et al., 2010; Gao and Jusko, 2012). The PK and PD of large molecules differ in several aspects from those of small molecules. For instance, the PK may depend on the PD in a process called target-mediated drug disposition (TMDD) (Gibiansky and Gibiansky, 2009). Understanding the factors that affect the PK of antibodies is of high importance for effective therapeutic application (Tabrizi et al., 2006). Several excellent review papers and books have been published on the topic of PK/PD modeling of antibody and protein therapeutics (Lobo et al., 2004; Wang et al., 2008; Meibohm, 2011).

### Samples for PK and PD

Ideally, samples for the PK and PD readouts are collected from the same animals. When collection from the same animals is not possible due to animal limitations, or due to the nature of the PD readout (e.g., where PD readout is perturbed by sample collection for PK purposes), a satellite group of animals may be employed. In such a situation it is important to match all aspects of the study design, e.g., gender, strain, species, dose, dose-administration, sample-times, disease state, and operator to minimize variability. In cases where the access to the disease model animals is limited and a satellite group is not available for PK sampling purposes, a “bridging experiment” in an alternate strain may be carried out to provide confidence in similarity of PK behavior between two groups. It is important to include a vehicle treated control group with drug treated groups when designing a PK/PD study. Changes in biomarker response after drug treatment are often obtained from comparison with control groups. Assessment of PD behavior in vehicle treated control group is critical in cases where biomarkers display circadian rhythm or when formulation vehicles are suspected to influence pharmacological effect.

### Sample numbers and time points

PK/PD analysis seeks to quantify drug concentration-pharmacological response-time relationships. In order to model PK/PD relationships, it is necessary to fully characterize drug concentration with time (pharmacokinetics) and modulation of PD effect with time (pharmacodynamics) after dosing. Sampling of data points should ideally allow detailed description of rise and decay of plasma concentrations as well as the onset, duration, and offset of PD response. The goal is to obtain well defined plasma exposures (AUC, C_max_, T_max_) and time to achieve maximum PD response, such that temporal delays between drug exposure and PD effects can be ascertained. It is recommended to collect samples up to and including time points where PD response has dissipated and returns to baseline, or matches that of control group.

Typical outputs of PK/PD models are E_max_ (maximum effect) and EC_50_ (drug concentration that causes 50% of E_max_) parameters, that define relationships of PD effect to drug concentration. E_max_ relates to intrinsic efficacy of drug and EC_50_ to its potency. Additional parameters, such as EC_20_, EC_80_ or EC_90_ may be useful that relate to concentrations that cause 20, 80, and 90% of the maximum effect, respectively. In order to obtain a reliable drug concentration-effect relationship and understand the magnitude of potential response, a robust PK/PD study design includes multiple dose levels such that adequate number of data points around a range of PD effects, including absence of effect, is obtained and at least one dose shows maximum PD effect. Ideally, but often not possible, adequate PD data will be obtained in the following three concentration ranges ≤ EC_20_, between ranges of EC_20_ and EC_80_, and at ≥ EC_80_. Once a PK/PD relationship has been established, it may be possible to refine sample collection based upon the understood relationship. However, care must be exercised when extrapolating findings between two different compounds, especially those from different chemical series.

### Selecting PD biomarkers

Biomarkers are factors that are objectively measured and evaluated as indicators of normal biologic processes or pathologic processes, and/or as indicators of pharmacological responses to therapeutic intervention (Colburn, 2003). When designing a PK/PD study it is important to consider the selection of the PD biomarker, and properties of the PD readout that could impact PK/PD correlations. One aspect to bear in mind is the proximity of PD biomarker or endpoint to the target and to the ultimate measure of efficacy. The measured PD response is ideally a direct measure of the target modulation; however, multiple steps frequently exist between the target and the biomarker being measured. Each of these steps would have a unique time-course of onset, duration, and offset of response that need to be considered to optimize the sampling design aspect of the PK/PD study. Other considerations prior to the start of a PK/PD study include whether *in vitro* and *in vivo* mechanisms are similar and whether the PD readout mimics the cell based assay. The target selectivity and specificity will ideally not impact the pharmacokinetics. Importantly, a biochemical link must exist between biomarker and disease state, and the dynamic range of biomarker response should relate to an efficacious readout in the animal model (Wang et al., 2008; Yamazaki et al., 2008). It is useful to examine if subtle changes in biomarker levels can be accurately and precisely captured, and whether the PD response after a single dose is predictive of the PD response after repeated dosing. In certain cases repeated dosing may lead to sensitization or tolerance of pharmacological effect, thus introducing dose- or time- dependent nonlinearity in biomarker response.

**Figure 3:**
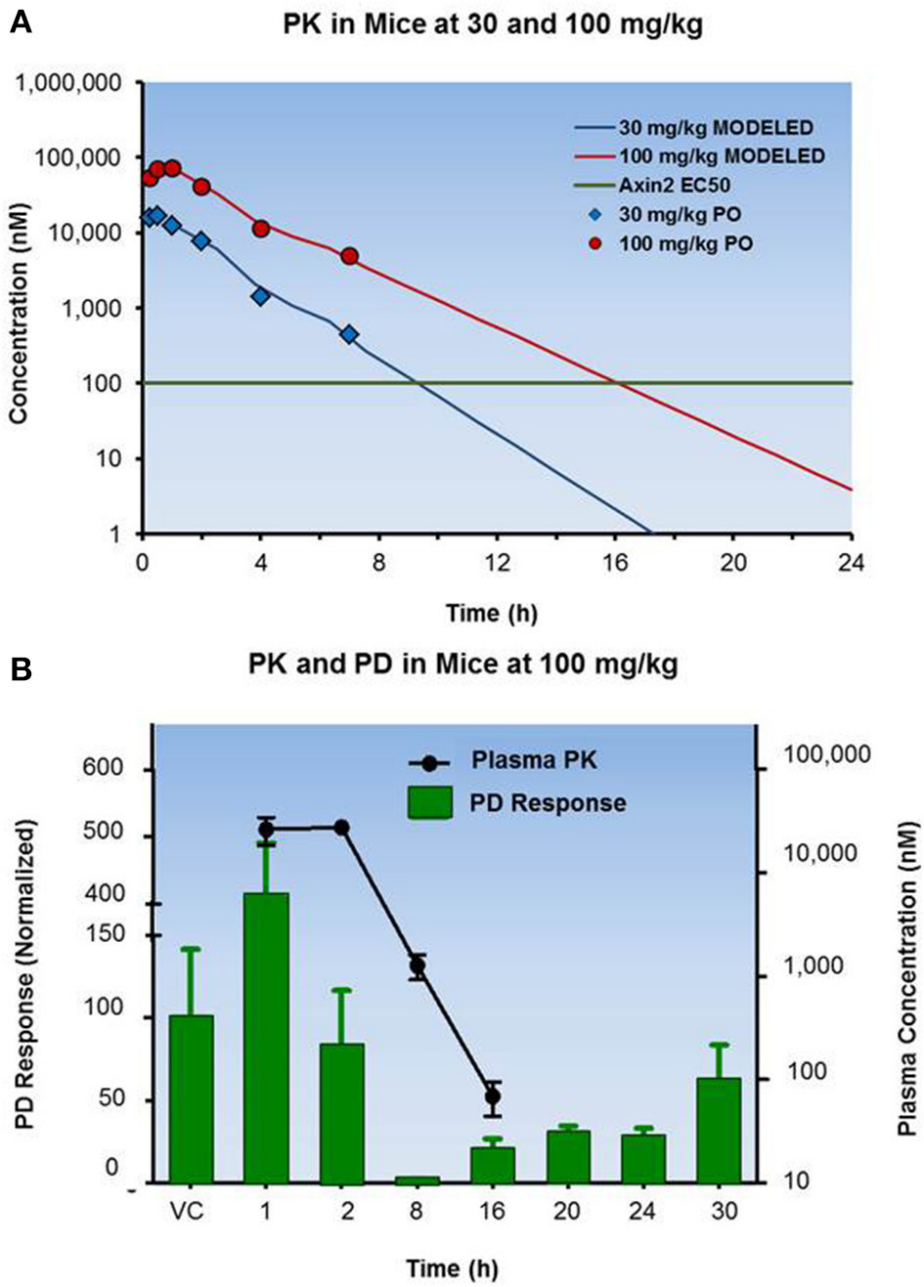
**PK and PD profiles of an oncology drug candidate in a mouse xenograph model**. **(A)** Displays the pharmacokinetic profiles of a compound following oral administration at 30 and 100 mg/kg to mice. **(B)** Illustrates the PK/PD results at 100 mg/kg with the drug showing return of the PD response to baseline.

The hypothesis that the team is testing when designing PK/PD sampling time points is worth careful consideration. In the recent case of designing PD sampling points for an oncology program, the team aimed to characterize the off-rate of pharmacodynamic response and hypothesized that the Axin2 mRNA (PD response) levels would return to baseline when the circulating concentrations of the drug candidate dropped below the Axin2 EC_50_. As a result, the team selected a rigorous sampling regimen of 16, 20, 24, and 30 h post dose to sample the Axin2 mRNA levels in the PK/PD study at 30 and 100 mg/kg (Figure 3). Only by including the later time points 24 and 30 h were the scientists able to observe the prolonged PD response followed by the gradual return of Axin2 mRNA response to baseline at the 100 mg/kg dose level.

Occasionally one may find that the PD effect influences the PK and vice-versa. For example, LXR agonists are known to have an agonistic effect on PXR due to very high homology between the two receptors, and consequently result in an induction of the CYP3A4 enzyme (Shenoy et al., 2004). If the drug is a substrate for CYP3A4, repeated administration of the test compound might result in altered pharmacokinetics.

### Unbound concentrations in plasma and tissue

It is desirable that PK concentrations and PD readouts are obtained from the same samples and animals, or matrix if using satellite animals. The preferred samples for PK/PD correlations are either blood or plasma. These matrices offer several advantages easy collection, straightforward approach to bioanalysis, and potential for translation across species through to the clinical setting.

Based upon theory of receptor pharmacology, it is unbound drug in blood or tissues that can interact with the target and elicit a pharmacological response (Smith et al., 2010). Once distributional equilibrium is achieved, assuming passive diffusion of compound throughout the body, the unbound concentration in plasma will reflect that in any given tissue. Therefore, it is recommended that colleagues consider unbound concentrations in plasma as a starting point for establishing PK/PD relationships. In situations where the PD readout is obtained from a different matrix (e.g., tumor, eye, brain etc.), and unbound tissue concentrations cannot be predicted from blood or plasma exposure, collection of PK information from tissue in addition to blood or plasma samples may aid in ultimate data interpretation. In these scenarios, PK/PD relationships may be derived from both target tissue and plasma exposure (Read and Braggio, 2010).

Whole tissue concentrations are often obtained from animals by homogenizing or lysing tissue and subsequently determining the drug concentration in the tissue homogenate. Common examples include tumors and brain which are collected primarily with the objective to get information about drug distribution to the target tissues. However, tissues are made up of distinct compartments (interstitial fluid, various cell types, various subcellular organelles) in which the drug is not necessarily distributed in a homogenous fashion. Moreover, total drug concentrations in tissue homogenates do not give any information about whether the drug is available for binding to the target receptor. Thus, when whole tissue concentrations are determined by measuring overall drug concentrations in the tissue homogenate, the concentrations found are not informative with respect to the pharmacologically active concentration of the drug at the site of action (Mouton et al., 2008).

**Figure 4:**
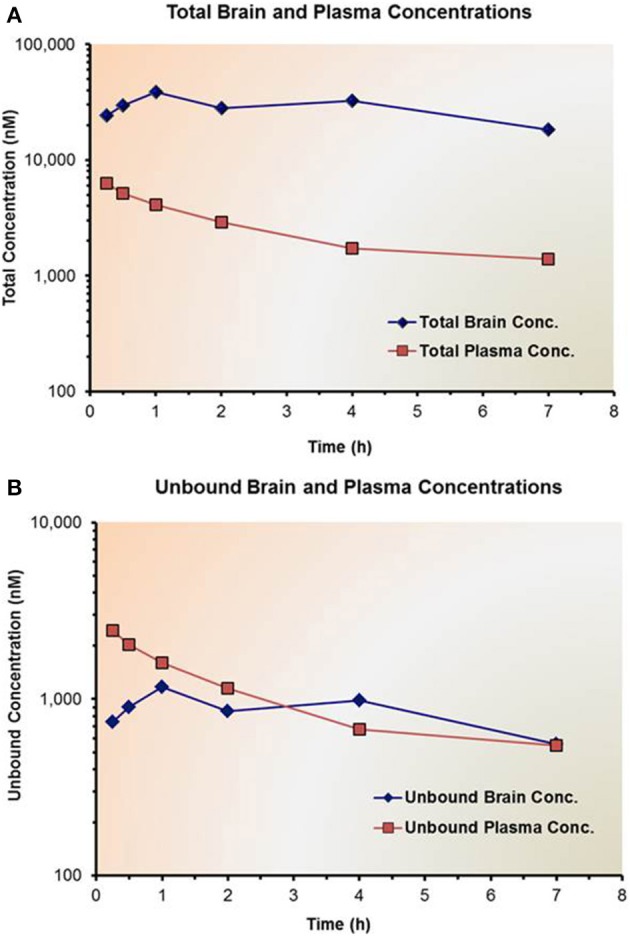
**Impact of non-specific binding on the total and unbound concentrations of a drug candidate in plasma and brain**. **(A)** Shows the total drug concentrations in brain and plasma, while **(B)** shows the corresponding unbound drug concentrations. Both plasma protein and brain homogenate binding studies were conducted using the Rapid Equilibrium Dialysis (RED) device.

Unbound target tissue concentrations might be useful to draw initial PK/PD correlations such as in brain. Unbound fraction of compound in target tissue can be estimated from *ex vivo* measurements in tissue homogenates using traditional techniques, such as rapid equilibrium dialysis (RED) in a similar fashion to measure unbound fraction in plasma (Banker and Clark, 2008). In other cases, *in vivo* unbound concentrations in tissues might be accessible using microdialysis techniques (Heinzen and Pollack, 2004; Raje et al., 2005; Kalvass et al., 2007). It is not uncommon that unbound concentrations in target tissues are in rapid equilibrium with unbound concentrations in plasma, which can then be used to establish and drive PK/PD relationships (Figure 4).

### Plasma protein binding assessments

Good experimental designs will take into consideration that unbound concentrations of compound may be responsible for driving the pharmacodynamic response. Although testing this hypothesis typically requires at least two compounds with different plasma protein binding (PPB) and PK profiles, fundamental principles of pharmacology would suggest that only unbound drug is capable of eliciting a pharmacological response. It is therefore important to incorporate some understanding of plasma and/or tissue binding of compounds into PK/PD study design and data interpretation (Trainor, 2007).

A current standard PPB measurement method in DMPK groups in the pharmaceutical industry is equilibrium dialysis using RED device (Waters et al., 2008). This assay is simple and straightforward and can be modified to permit determination of compound binding to homogenate from various tissues (e.g., a surrogate for tissue binding). When conducting binding studies, the compound must have adequate solubility in the assay buffer to eliminate any artifacts of poor physicochemical properties on the assay results. Species differences exist in the abundance of alpha-aminoglycoprotein (AAG) between rodents (2 μM) and humans (20 μM). *In vivo*, protein binding in disease models and disease states can differ from protein binding measured *in vitro*. When changes in binding are suspected, measuring *ex vivo* PPB or in plasma collected from animals is suggested.

*In vitro* potency measurements are often assessed in the presence of serum or plasma. If an *in vitro* assay medium contains plasma or albumin, apparent IC_50_ (or EC_50_) may be affected by compound binding to such proteins. As a first step, it is recommended to consider compound potency in the absence of serum/protein while taking into consideration the unbound fraction as determined in a separate *in vitro* PPB study. Rarely is there value in assessing a serum/protein-shifted potency in conjunction with total plasma concentrations to establish a PK/PD relationship (Smith et al., 2010).

### Simulating the exposure—effect relationship

Observations of PK exposure after a specific single dose can be utilized to predict the exposure after administration of a different dose or after repeated dosing. For example, the concentration-time data from a single oral dose at 10 mg/kg in rat can be used to simulate the PK exposure after repeated administration of 20 mg/kg twice daily (BID) for 7 days in the same animal model. The goal of the simulation is to predict a reasonable dose and dosing frequency that would result in desirable PK exposure and consequently a measurable biomarker or efficacy response. Depending on the target and whether the animal model is acute or chronic, the recommended dosing regimen could consist of a single dose or multiple doses that would produce steady-state blood concentrations.

**Figure 5:**
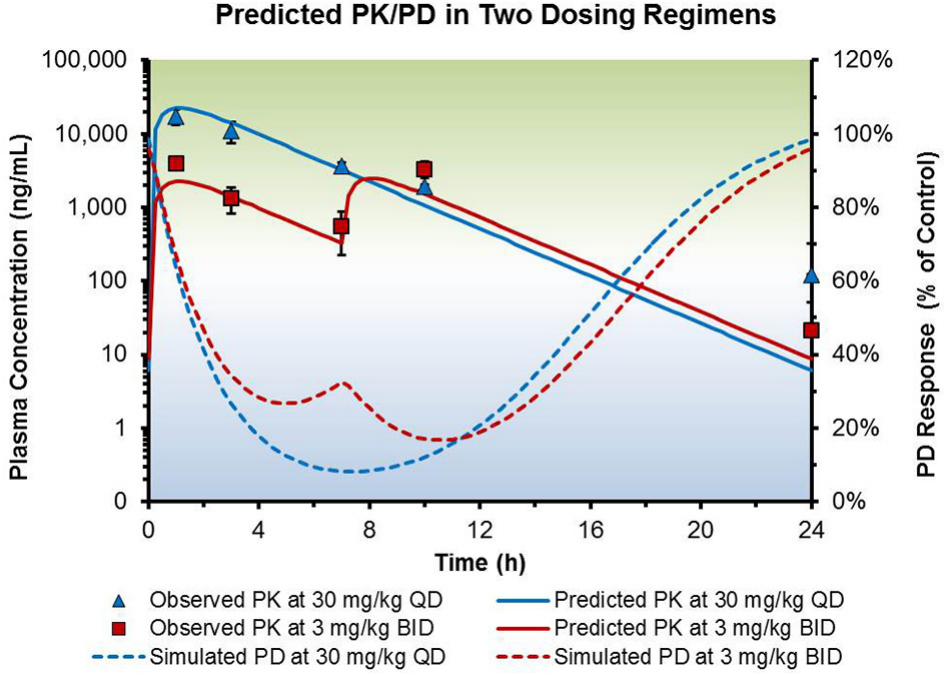
**Simulation of steady-state exposure and efficacy data of hypothetical dosing regimens in a preclinical mouse model**. A drug was dosed at 30 mg/kg once daily (QD) or 3 mg/kg twice daily (BID) in a mouse xenograph model. Observed and predicted plasma levels were plotted with the simulated PD responses to aid selection of dose and dosing frequency in a follow-up efficacy study.

Once exposure and efficacy data from a preclinical animal model using a specific dosing regimen are fitted in a suitable PK/PD model, the fitted parameters can be used to simulate exposure and efficacy using different dosing paradigms. In the example below, steady state exposure and percent inhibition of biomarker response of a test compound was simulated from early preclinical data. In the protocol planning phase before conducting elaborate chronic studies, the team simulated the exposure and PD responses using a variety of hypothetical dosing regimens. The simulations enabled the team to compare and contrast the impact of dose and dosing frequency on exposure and response changes. Consequently, the team was able to make informed decisions regarding selection of dose and dosing frequency in the chronic study, which ultimately increased the probability of obtaining the desired efficacy outcome (Figure 5).

Gao and Jusko used simulations to predict the maximum insulin responses in rats after glucose challenge with various infusion rates of glucacon-like peptide 1 (GLP-1) analog (Gao and Jusko, 2012). Simulations of exposure and efficacy in preclinical models may help select the best molecule from multiple compounds of same class. For example, simulations of time course of concentration at the effect site and pharmacological response enabled investigators to differentiate between three similar compounds, and results obtained in subsequent clinical trials confirmed the simulation results (Miller et al., 2005). Investigators at Novartis used simulations of exposure and efficacy from a rat model to predict human PK and PD of new drug candidates (Heimbach et al., 2009). The predicted human oral plasma concentration time profile was in good agreement with the observed clinical data, and rat PD effect parameters were predictive for human PD.

### Dose fractionation studies

The observed pharmacodynamic effect in a given preclinical model system is associated with a specific pharmacokinetic driver such as parameters AUC, C_max_, or C_min_ compared to the *in vitro* potency measure. In order to examine the pharmacokinetic driver for efficacy, one could consider dosing strategies to discern the relationship between different non-compartmental PK parameters relative to the observed pharmacodynamic effect. However, for compounds with linear pharmacokinetics, changes in dose alone will result in equivalent changes in each of the pharmacokinetic exposure parameters. Thus a two-fold higher dose will result in two-fold increase in both C_max_ and AUC rather than a differential change in which one PK parameter shows superior correlation with the efficacy readout. Therefore, the PK driver can't be determined by changing the dose alone and the driving exposure parameter may remain elusive even after extensive preclinical PK/PD profiling.

**Figure 6:**
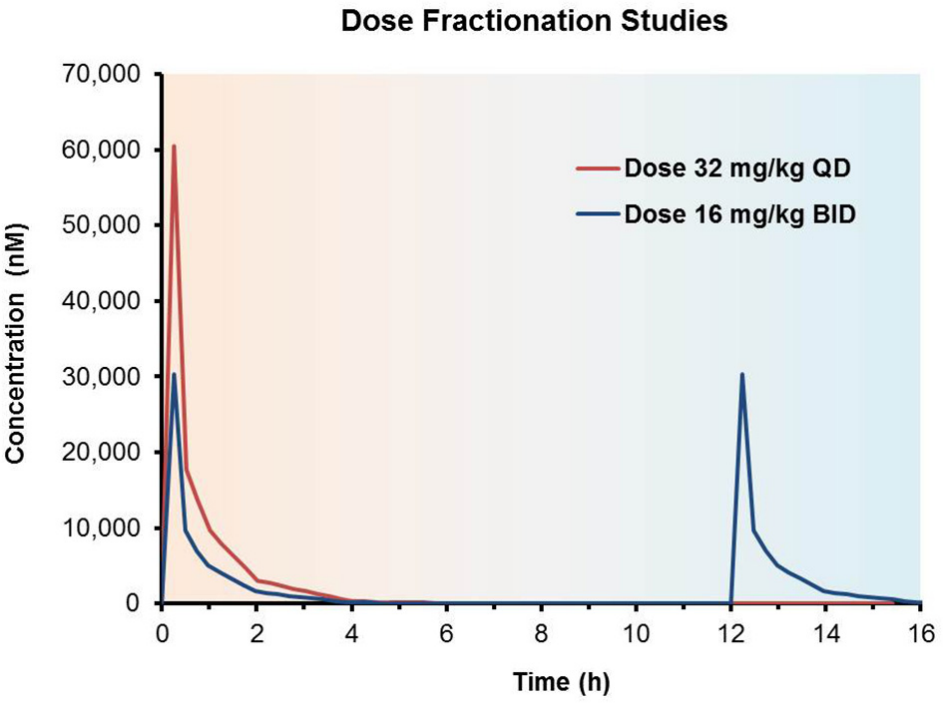
**Dose fractionation studies to determine the pharmacokinetic driver**. Impact of once daily (QD) vs. twice daily (BID) dosing regimens of the same total daily dose; both doses yield identical overall AUC_0-24 h_ values but different C_max_ concentrations over the course of the dosing regimens.

Interestingly, by fractionating the dosing intervals throughout the course of a given time frame, e.g., once daily (QD) vs. twice daily (BID), one can design studies that result in identical AUC over a given time interval but that has an altered C_max_ and/or C_min_ when comparing different dosing regimens. For example, a dose split into half the dose given twice over the same time interval will have the same AUC (34 h^*^nM) but half the C_max_(30,000 vs. 60,000 nM, Figure 6).

When done at several dose levels, each with two or three dosing regimens and combined with PD/efficacy, this method may elucidate the exposure effect relationship for a compound in a given model system and enable both identification of an optimal “target” PK profile, as well as significantly streamline future PK/PD study design. Although this approach is somewhat resource intensive, the power of the data that a study of this nature provides can bring major impact to a project. Investigators at Genentech used dose fractionation studies to examine the driver of efficacy for anti-tumor activity of T-DM1. By systematically varying the dose, dosing frequency and treatment duration in a mouse xenograph model they were able to demonstrate that the antitumor activity of T-DM1 is both concentration and time dependent, i.e., AUC is the PK driver of efficacy (Jumbe et al., 2010).

Antimicrobial agents can be categorized on the basis of the PK/PD measure that is most predictive of efficacy (Craig, 1998; Andes and Craig, 2002). Three common PK/PD measures of antimicrobial activity in preclinical infection models are the duration of time a drug concentration remains above the minimal inhibitory concentration (T>MIC), the ratio of maximal concentration to the MIC (C_max_:MIC), and the ratio of the area under the concentration–time curve at 24 h to the MIC (AUC_0−24 h_:MIC). Various antimicrobial agents including aminoglycosides and quinolones have been shown to have concentration dependent (AUC_0−24 h_:MIC or C_max_:MIC) efficacy, while penicillins and tetracyclins were demonstrated to have time dependent (T>MIC) efficacy (Ambrose et al., 2007).

## Data analysis and interpretation of PK/PD studies

Once DMPK and pharmacology colleagues have designed and executed a PK/PD study and the sample analysis has been completed, the team is left with a dataset from both PK and PD aspects of the study. A thorough analysis of such data will help to understand the mechanism of action of the drug, to compare different compounds, and to help select compound for progression to further development.

### Correlation of concentration and effect

The initial approach to evaluate the correlation of the concentration vs. time (PK) and effect vs. time (PD) profiles is through visual representation. By plotting effect vs. concentration and removing the time variable, the relationship between PK and PD will manifest as either a direct (instantaneous) or indirect (temporal delayed) relationship. In order to represent the connection between PK and PD adequately, careful preparation in the study design phase of the experiment will enable a pharmacological measurement in conjunction with a PK sampling point. In the case of instantaneous equilibrium, plotting effect vs. concentration will yield a linear, or non-linear, relationship such that increasing concentration yields an increasing effect and decreasing concentration will result in a decreasing effect. When concentration ranges obtained cover a wide range, the relationship may better be described by a log (conc)-linear (effect) plot to better describe the PK/PD.

Often, the same concentration can yield different pharmacological responses. This phenomenon is referred to as hysteresis, which can be characterized as either clockwise or counterclockwise depending upon the directionality of time in concentration relative to response. By plotting concentration vs. effect and observing hysteresis, one can not only hypothesize as to the underlying biological mechanism driving response, but also can select subsequently an appropriate PK/PD model to “collapse” the hysteresis loop and estimate parameters that describe the concentration-effect relationship. Several textbooks provide excellent summaries of various PK/PD models and equations to support the analysis of linear, log-linear, sigmoidal E_max_ and E_max_/I_max_ models as well as PK/PD relationships that show hysteresis (Gabrielsson and Weiner, 2007; Rowland and Tozer, 2010).

### Selecting appropriate PK/PD model

As highlighted above, plotting the effect vs. concentration data will yield a relationship between PK and PD and provide insight as to appropriate PK/PD model selection for subsequent data analysis. Although the ultimate choice of model will often be dictated by the available data set, analysis will generally fall into two different categories: instantaneous effects and temporal delayed effects. Strategies, considerations, and examples of applications of PK/PD models in drug discovery and early development have been presented in several outstanding review papers (Derendorf and Meibohn, 1999; Derendorf et al., 2000; Gabrielsson et al., 2009, 2010; Amore et al., 2010; Van der Graaf and Neil Benson, 2011; Visser et al., 2013).

#### Instantaneous effects

When compounds achieve rapid equilibrium with the biophase and effects are directly mediated by drug concentrations, PK/PD relationships can be characterized with models ranging from simple linear models to more complex sigmoidal E_max_ models. All of the models can incorporate a baseline response but simple linear, log-linear, and exponential models operate under the premise that the effect is not limited, e.g., increasing concentration will always increase a response. Comparison of effect vs. concentration plots where concentration is plotted on a linear vs. logarithmic scale as well as an apparent lack of maximal effect can be valuable in helping confirm the selection of these models.

In cases where the pharmacodynamic response asymptotically approaches a maximum effect, an E_max_ model will likely better describe the data set. When effect-concentration data are plotted on a linear-log scale, data appear as a sigmoidal shape with a maximal effect that shows a plateau. Often referred to as the Sigmoidal E_max_ model, the slope of the relationship can be better characterized by applying a shape factor (or Hill coefficient, ϒ) in the mathematical description of the data. When the Hill coefficient is equal to unity (ϒ = 1.0), the equation below is reduced to the Simple E_max_ model.

(1) $$\text{Effect}={\text{E}}_{0}+\frac{{\text{E}}_{\text{max}}•{\text{C}}^{\gamma }}{\text{E}{\text{C}}_{50}^{\gamma }+{\text{C}}^{\gamma }}$$

**Equation 1:** Sigmoidal E_max_ equation where E_0_ is the baseline response, E_max_ is the maximal response, C is the drug plasma concentration, ϒ is the Hill coefficient, and EC_50_ represents the concentration at 50% response.

Although in the context of *in vivo* data, the shape factor is purely empirical and lacks any *in vivo* relevance, the use of the Hill coefficient can significantly improve the model fit to the data. In cases where several receptor systems work together or compete to drive ultimate PD response, composite I_max_/E_max_ models (variations of fundamental E_max_ models) can be applied to describe more complex direct concentration-response relationships.

**Figure 7:**
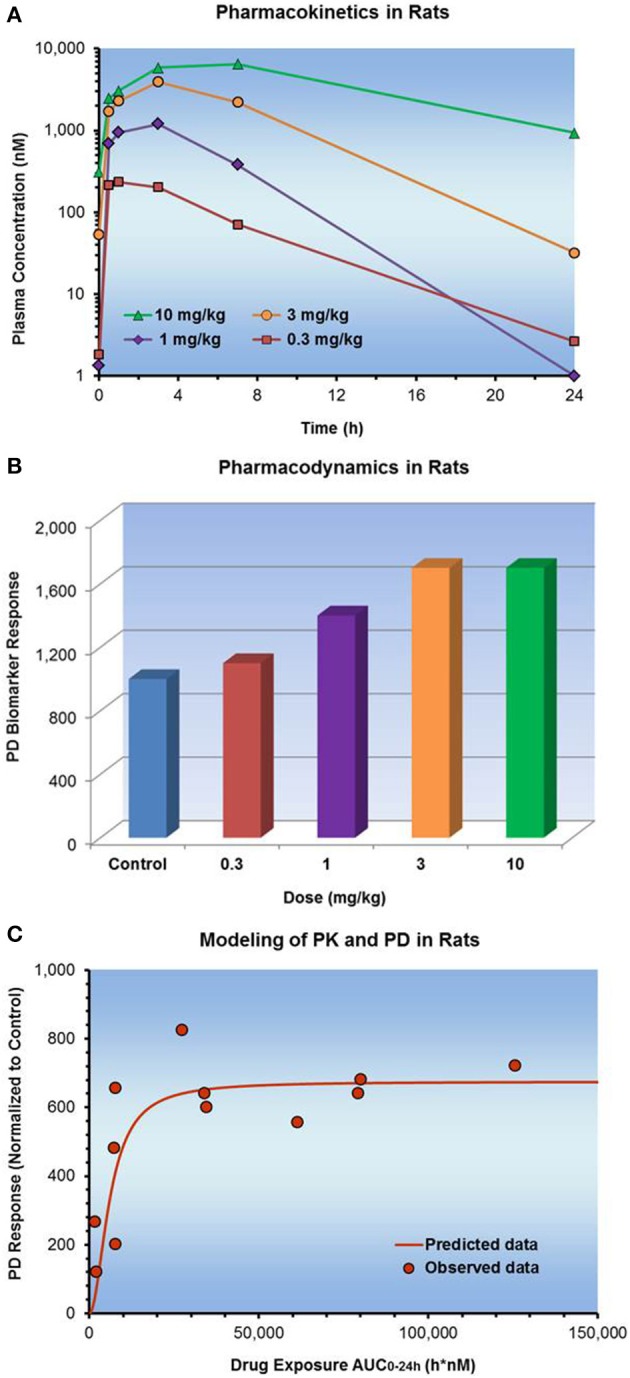
**Dose dependent PK and PD observed in a rat model of diabetes**. **(A)** Shows the PK with time, **(B)** shows the PD with time, and **(C)** plot of PK vs. PD. There was instantaneous equilibrium between exposure and effect, thus the PK/PD data were modeled using a direct Sigmoidal E_max_ response model.

In a hyperglycemic clamp study in a rat model of diabetes, a test compound was administered orally and glucose was infused simultaneously at a variable rate to raise and maintain blood glucose concentrations at approximately twice the level of baseline blood glucose levels. The glucose infusion rate (GIR) needed to maintain the glucose level correlated with the systemic exposure of a test compound (Figure 7). By plotting PK exposure (x-axis) vs. PD response (y-axis) on a log-linear scale, a direct and time independent relationship of PK and PD data became apparent. At high systemic exposures, the PD response was approaching an asymptotic maximum value, E_max_. These PK/PD data were modeled successfully using the Sigmoidal E_max_ direct response model.

#### Temporal delays in effects (Hysteresis)

Relative to the time course of pharmacokinetics, the pharmacodynamic effect can be delayed or shifted for several reasons. Distributional delay to the target site, indirect action, kinetics of receptor activation, active metabolites, and changes in baseline over time all represent underlying mechanisms that can manifest in delayed effects. Models to fit to data sets that show disconnects between plasma concentration and pharmacological effect include PK/PD link models (Jusko and Ko, 1994), indirect response models (Jusko and Ko, 1994; Salphati et al., 2010), and receptor-based models (Ploeger et al., 2009). Selection of appropriate models relies upon visual inspection of the data as well as insight on the underlying pharmacokinetics and pharmacology. Fundamental assumptions behind the PK/PD link model are that the delay in response is dictated by distribution to the effect site, that loss of effect is driven by loss of compound from the biophase, and that re-input of compound from the biophase back into plasma is negligible.

The premise behind turnover models (also known as indirect response models) is that the compound does not elicit a direct response; rather it acts to either stimulate or inhibit the onset or offset of response. As a result, four different relationships have been established: stimulation or inhibition of turnover rate (K_in_) and stimulation or inhibition of fractional turnover rate (K_out_). Understanding the mechanism of action and baseline turnover rates can aid in both model selection and establishment of initial parameters for modeling (Danhof et al., 2008).

**Figure 8:**
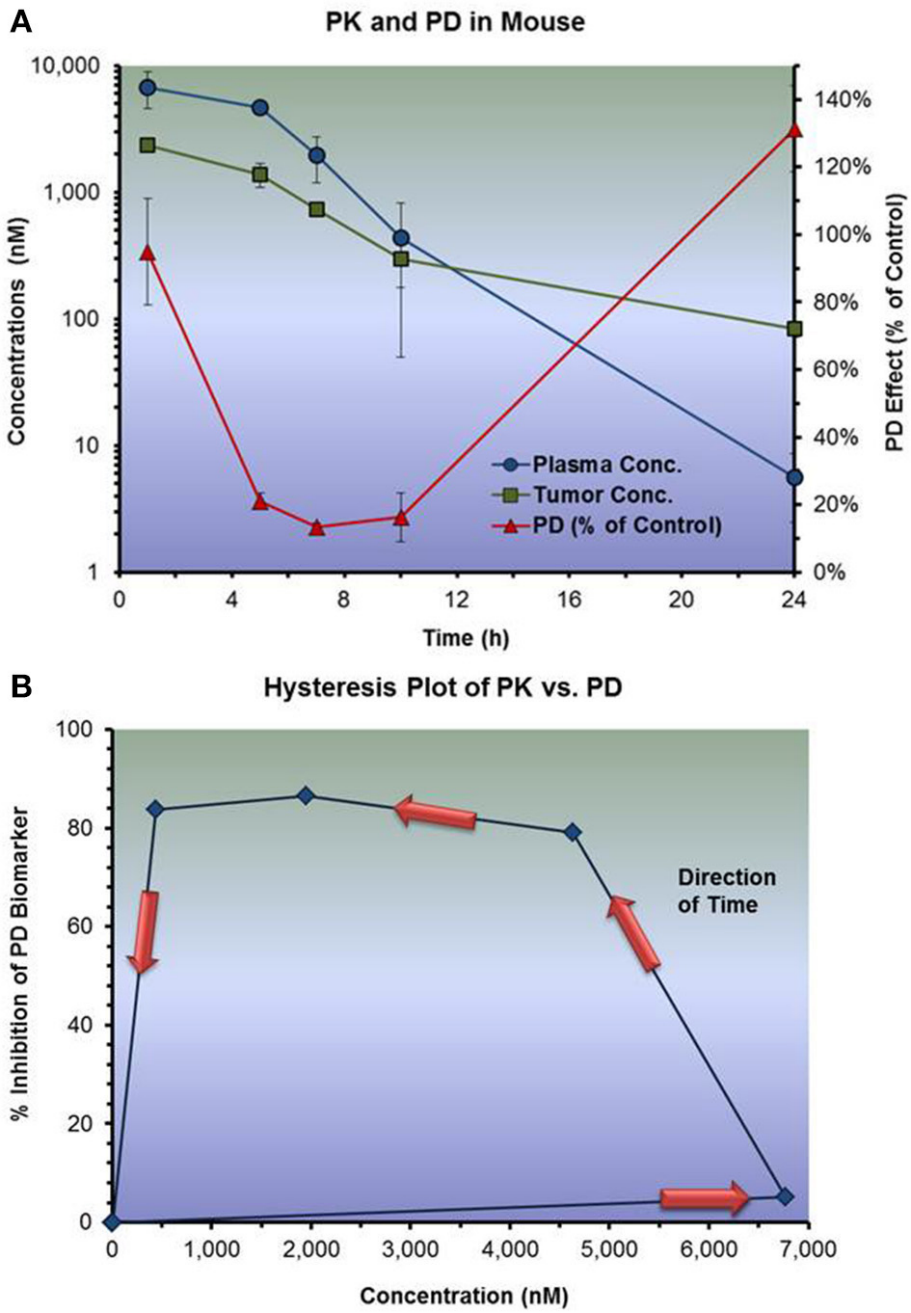
**Effect vs. plasma concentration of a drug candidate following a single oral dose to tumor bearing mice**. **(A)** Shows the PK and PD response both plotted against time. **(B)** Shows the PK plotted directly against PD. Delayed pharmacodynamic response resulted in a hysteris plot. An indirect response model (inhibition of input function) was subsequently developed to describe and predict PD response inhibition based upon various dosing regimens.

In the example below, temporal delay in inhibition of tumor biomarker response was observed for oncology drug candidates when administered as a single oral dose to tumor bearing mice (Figure 8). While the plasma concentration peaked one hour post-dose, maximal inhibition of the biomarker response was observed at 8–10 hours. When plotting exposure vs. response, a hysteresis plot emerged in which concentration and response were time dependent. The arrows in the counter-clockwise hysteresis plot represent the directionality of time throughout the course of the experiment. Equipped with knowledge about the target and presumed mechanism of action of the drug, the PK/PD modeler applied a turn-over model (indirect response model) to this data set to get an estimate of EC_50_.

Complex PK/PD relationships such as peak shifts, a change in baseline response, transduction, synergy, and tolerance will require modifications to turnover models to fully characterize these datasets (Mager et al., 2003). Mechanism based PK/PD models can provide improved insight in drug actions and effect, such as to describe the phenomenon of TMDD of biotherapeutics (Gao and Jusko, 2012). The time course of the therapeutic effect of anticancer agents in mouse xenograph models is often delayed relative to the systemic exposure. Modeling the complex time course and temporal delay of anticancer agents was successful by introducing a series of transit compartments that were related to a cascade of kinetic events that yield drug effects (Lobo and Balthasar, 2002). Tumor growth kinetics were included in similar models to characterize tumor growth inhibition of anticancer drugs (Simeoni et al., 2004; Bernard et al., 2012), while a semi-mechanistic transit compartment tumor kill PK/PD model was used to describe anti-tumor activity of Trastuzumab-DMI (Jumbe et al., 2010). Finally, target binding kinetics may be an important consideration for compounds that bind tightly to the receptor target. The duration of effect of compounds exhibiting slow target binding dissociation kinetics can be highly correlated with the drug-target residence time (Dahl and Aherud, 2013). The PK/PD modeling of such drugs, particularly if the drug binds covalently to the target, should incorporate dissociation rates from the target and/or turnover rates of the biological target itself.

The models described above provide a context for the general types of models that can be applied to PK/PD datasets. Ultimate factors impacting the selection of an appropriate model include the quality of the data and the richness of the dataset, the success of the study design to adequately capture the entire concentration and effect vs. time profiles, the fundamental understanding of the biological system or animal model, and statistical and mathematical output. Output from the model including visual inspection, evaluation of residuals, and statistical analyses are critical factors that drive final model selection. Other more complex models that support instantaneous equilibrium (e.g., power function, biphasic, composite models, etc.) can be found in a textbook titled “Pharmacokinetic and Pharmacodynamic Data Analysis: Concepts and Applications” (Gabrielsson and Weiner, 2007).

### Relevance of *in vitro* potency to *in vivo* plasma/tissue levels

In theory, if an *in vitro* system completely mimics the *in vivo* environment with respect to target interaction, concentrations needed to elicit an effect *in vitro* (e.g., EC_50_) should manifest into *in vivo* response at an equivalent effective plasma or tissue concentrations. Implicit in the validity of this relationship are several pharmacokinetic assumptions, including the lack of a distributional barrier between the target and the site of measurement (e.g., plasma), the lack of differences in binding *in vitro* vs. *in vivo* (e.g., PPB, non-specific binding), as well as linearity of pharmacokinetic disposition over the relevant concentration range. In addition, multiple pharmacodynamic assumptions such as the absence of any response from non-specific pathways, absence of response from non-target tissues, and the lack of any target manipulation (e.g., tolerance development, sensitivity) that might not be captured *in vitro* when moving to an intact *in vivo* model need to be considered. Given these challenges when moving from a cell-based system to the complex *in vivo* animal, it can't be expected that *in vitro* potency will translate directly into *in vivo* potency. However, occasionally this direct relationship may hold and one may be able to leverage this observation to develop subsequent hypotheses as well as possibly validate the above assumptions.

**Figure 9:**
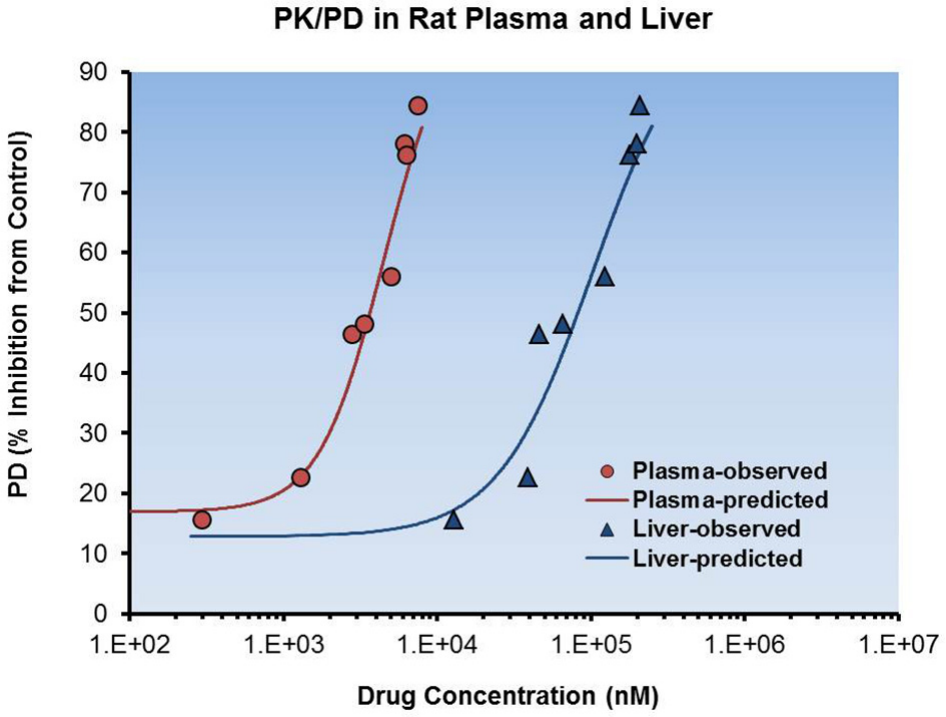
**Total plasma and liver exposures relative to pharmacodynamics response**. Rat total plasma and target tissue (liver) exposure was plotted with PD effect. A sigmoidal E_max_ model with baseline and Hill coefficient (γ) correction factors was used to describe the dataset. Improved *in vitro-in vivo* correlation was obtained when converting total plasma concentration to free plasma concentration for the estimate of *in vivo* EC_50_.

In the case of the inhibition of a lipid biosynthesis target in the liver, the project team sought to establish a PK/PD relationship between plasma or liver concentrations and the percent inhibition of a liver pharmacodynamic marker in rats. Figure 9 shows the fit of a Sigmoidal E_max_model to the data in both liver and plasma. From these data the team was able to determine an *in vivo* EC_50_ and then compare to the *in vitro* potency observed in the HepG2 cell based assay. When total concentrations were compared, plasma EC_50, *in vivo*_ (4.5 μM) was much higher than expected from the *in vitro* data (0.7 μM). However, when adjusted for PPB, EC_50_ values were similar (0.9 vs. 0.7 μM). From this observation, the team was able to hypothesize that unbound concentrations in plasma of discovery compounds when normalized for *in vitro* potency might enable streamlined PK/PD study design in the future and eliminate a need to assay any liver concentrations of compounds.

### Binary response data

In some cases due to experimental limitations or lack of a priori considerations for study design, PK/PD data are obtained where the response is a simple conclusion of “Effect” or “No effect.” In other situations, the output of a study may be efficacy wherein the response is not graded but categorical (e.g., death vs. survival). In these cases, further interpretation is often difficult due to the limited response dataset and detailed PK/PD modeling may not be possible. However, some considerations from a pharmacokinetic and pharmacodynamic viewpoint are helpful, such as arriving at a study design that can achieve adequate exposure in the animals to elicit the desired PD response. Based upon the time needed to achieve the endpoint, subsequent studies may be designed to better understand the dynamics of response and the relationship to concentration. An important goal of such studies is to be able to differentiate compounds based upon the results. A mechanistic hypothesis as to why some compounds showed a response vs. others that didn't is beneficial at this stage. One may consider a dose fractionation approach or altered dosing regimen help to define the pharmacokinetics needed to achieve the endpoint.

Modeling behavioral pharmacology of psychotropic drugs is often complicated by the fact that non-continuous pharmacodynamic endpoints are reported on a categorical scale (Geldof et al., 2007). Proportional odds models have been successfully applied to data from analgesic trials in which severity of pain, albeit a continuous measurement, is recorded on a categorical scale and used at the pharmacodynamic measure (Nestorov et al., 2001; Bender et al., 2009).

On a quest to discover and develop a new class of anti-malaria drugs, Novartis scientists studied efficacy in a malaria infected mouse model (Meister et al., 2011). The team used measurements of parasitemia reduction and days of survival as pharmacological endpoints. Compound GNF179 reduced Plasmodium Berghei parasitemia levels by 99.7% with a single 100 mg/kg oral dose and prolonged mouse survival by an average of 19 days (Table 1). In contrast, average survival times for Chloroquine and Artesunate in the same model at 100 mg/kg single dose were 12 and 6.7 days, respectively. While “days of survival” is a semi-quantitative measure of efficacy, it is a categorical endpoint that can't easily be modeled. By rank ordering compounds according to percent parasite reduction and survival length at multiple dose and exposure levels, the team was able to identify an anti-malaria drug candidate for clinical development.

**Table 1:** **Parasitemia reduction and survival in a Plasmodium Berghei mouse model of Malaria infection (Meister et al., 2011)**.

	**Dose mg/kg p.o.**	**Animals tested**	**Parasitemia reduction (%)**	**Survival (days)**
Untreated	n/a	10	0	6.5
GNF179	1 × 100	3	99.5	19.0
Artesunate	1 × 100	>10	97	6.7
Chloroquine	1 × 100	>10	>99.9	12

In preclinical models of Hepatitis C viral replication, the endpoint in the study is a 1-log reduction in viral replication. To gain insight on the PK/PD relationship of inhibiting viral replication, studies are often designed over a dose range, with altered dosing regimens, or by leveraging historical results with compounds that have demonstrated efficacy (Kamiya et al., 2010). This integrative process provides a better understanding of the mechanism of drug action, suggests improved animal models to evaluate drug targets and drug-disease interactions, and helps to design animal experiments that provide more clinically useful information. Furthermore, it allows investigators to predict drug class liability with respect to safety, and generate exposure-response relationships for efficacy and safety which can be extrapolated from animals to humans. Translation to the clinic with binary data should be made with caution, especially in the absence of robust dose response.

### Multiple biomarker data

Access to a well characterized drug target and biological pathway(s) involved in modulation of disease is a tremendous advantage when selecting biomarker(s). In cases where disease pathways are well-understood, collaboration and input from the developmental-molecular pathways and modeling-simulation groups may aid in quantitatively modeling the pharmacodynamic cascade and may expedite PK/PD model development.

When presented with several potential biomarkers, teams are asked to qualify the validity of one biomarker over another and select the most appropriate PD endpoint for analysis (Vaidya et al., 2010). In some cases, teams may have the ability to monitor multiple biomarkers within a single PK/PD study. Although this may aid in a more comprehensive capture of the biological response, it may also complicate the data analysis and provide limited additional insight toward subsequent study design. A consideration of the experimental question being asked (e.g., prove target engagement vs. demonstrate a pre-clinical endpoint such as reduction of tumor volume) is key for proper selection of a biomarker relevant to the study purpose. Scientists from Astra-Zeneca have outlined a biomarker classification (e.g., nomenclature) system for use during target validation, lead generation, lead optimization and candidate selection stages (Visser et al., 2013).

**Figure 10:**
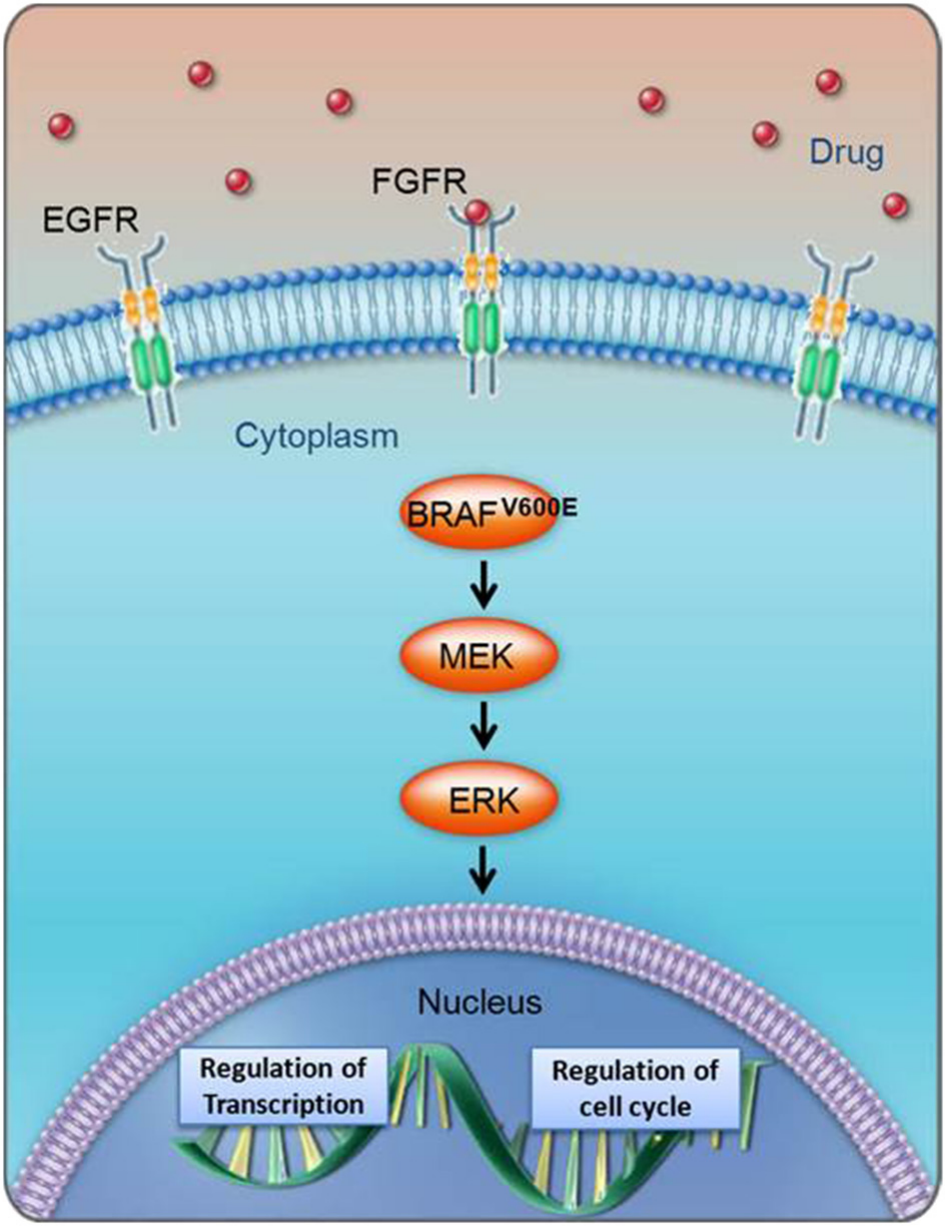
**Biological cascade of BRAF^V600E^ activation and cell cycle implications**. The BRAF^V600E^ pathway includes multiple biomarkers whose direct or indirect response could be indicative of efficacy.

It is known that normal cells can convert to cancer cells when mutations occur in genes that control kinase signaling cascades and thus regulate cell proliferation and differentiation. For example, more than 40 different mutations have been identified in the BRAF gene in human cancer. A change at residue 600 in the BRAF gene (V600E) leads to 500-fold increased activation of BRAF^V600E^-MEK-ERK signaling in tumor cells, a signaling pathway that is frequently mutated in melanoma (Cantwell-Dorris et al., 2011). Pharmaceutical scientists have relatively recently developed anti-cancer agents that target specific kinase signaling pathways relevant to tumorigenesis. Small molecule BRAF-specific inhibitors block the kinase activity of BRAF^V600E^, thus preventing it from activating its downstream targets and subsequently inhibiting tumor cell proliferation (Figure 10).

When presented with multiple biomarker data, it is important to identify the rate-limiting steps that might be driving the response of a given biomarker. The closeness of the drug target to the biomarker in a signaling cascade will impact the data interpretation. In the case of the BRAF program, a Novartis team was able to measure both phospho-MEK and phospho-ERK as biomarkers to indicate modulation of the BRAF^V600E^ pathway. Based upon these results, the model to describe the effect-concentration relationship between p-ERK and plasma vs. p-MEK and plasma was evaluated. Assuming the goal of the study is to understand target engagement in BRAF, the phospho-MEK:plasma analysis could enable subsequent study design around better understanding of the duration of response and decline of the phospho-MEK signal. Alternatively, if one attempts to understand the relationship to the potential for tumor shrinkage in a follow up efficacy study, the phospho-ERK:plasma analysis might better enable this.

**Figure 11:**
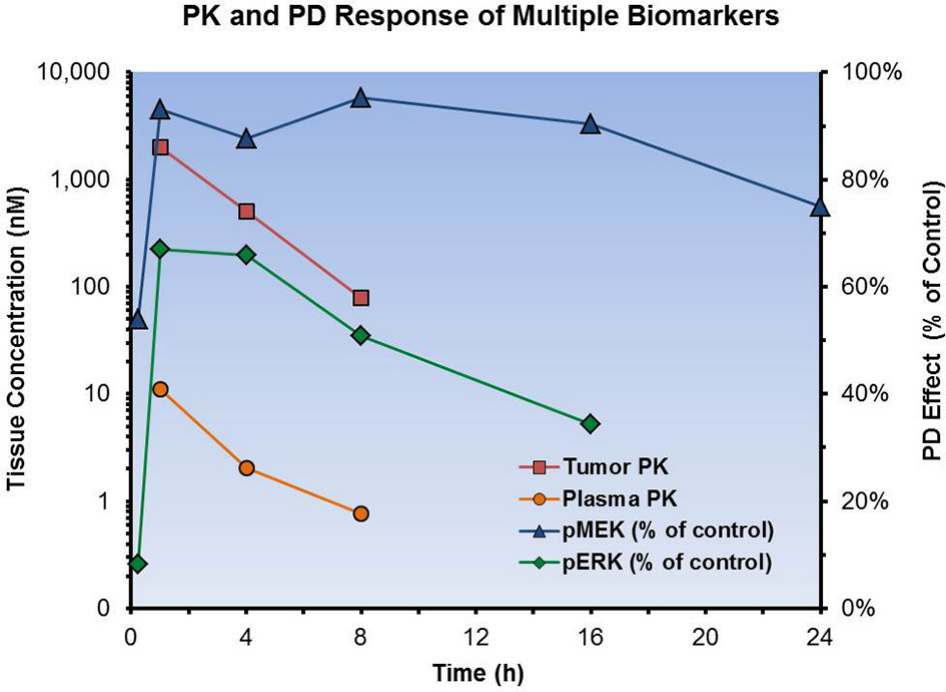
**Using multiple biomarkers to indicate modulation of the BRAF^V600E^ pathway**. A single oral dose was administered to tumor bearing mice. Plasma and tumor tissue were collected from the same mice to simultaneously obtain plasma PK and biomarker response of phospho-MEK and phospho-ERK in tumors.

Figure 11 shows a depiction of PK/PD data of multiple biomarkers in the BRAF^V600E^ pathway where one can observe time delay between plasma peak concentrations and maximal biomarker response. As a result the project team decided to use a PK/PD-link model to further describe the data and characterize the PK/PD relationship.

As outlined in the earlier Section “Selecting Appropriate PK/PD Model,” modeling of the time-dependent signal transduction (i.e., the signaling cascade) of anticancer agents may be accomplished using extended versions of indirect response models (Lobo and Balthasar, 2002; Simeoni et al., 2004; Jumbe et al., 2010; Bernard et al., 2012). Several pharmaceutical companies have published sophisticated preclinical PK/PD models that successfully characterized plasma and tumor concentrations, tumor pharmacodynamics and antitumor efficacy of multiple promising anti-cancer drugs (Yamazaki et al., 2008; Wong et al., 2011, 2012; Marsilje et al., 2013).

### Variability in PK and PD

When fitting a model to pharmacokinetic and pharmacodynamic data, variability associated with PK and PD data within each study is a possible concern. When large variability in measured PK/PD data is expected or observed, the use of pooled data from different studies can help define the mean relationship between concentration and effect. In other cases it may be valuable to understand the reason for variability in the PK (e.g., different absorption profiles in different animals) and PD (e.g., variability in the tumor expression of target between animals) to better understand the underlying biology. Factors that could impact the variability are the number of animals (“*n*”) adequate to best capture the exposure and response data. When planning the PK/PD protocol, it may be helpful to collaborate with a statistician to ensure the study will have sufficient power to achieve statistically significant results.

When assessing the impact of variability in a dataset, it is important to consider the dosing and sampling strategy used for PK and PD measurements. If the PK and PD were collected from different animals within the same study (e.g., using a satellite group for the PK sampling), the variability may be reduced compared to a study where PK and PD data were generated independently of each other. In some cases variability is introduced as different dosing regimens are employed to assess PK/PD data from several individual studies, or PK sampling captures a limited portion of the overall pharmacokinetic disposition of the compound (e.g., 1–2 time points).

In any of these cases data analysis can still be conducted. However, it is important to identify when discussing and presenting the data that the results reflect multiple studies, and to interpret such data with the caveats of the study design. Variability can impact not only the quality of the data and the selection of the relevant PK/PD mathematical model, but also the interpretation of the resulting PK/PD relationship. If data from multiple studies and animal models are combined to support the modeling, the resulting parameter values may not be single point estimates but rather a distribution of parameter values generated from the uncertainty in the parameters estimated from the preclinical data (Chien et al., 2005).

**Figure 12:**
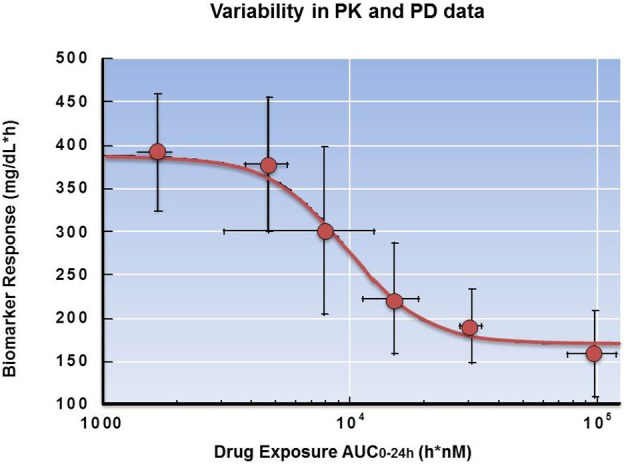
**Variability in PK and PD data**. Dose-dependent suppression of biomarker response in rats following administration of a drug candidate. The error bars represent variability in both plasma AUC and biomarker response.

In Figure 12 the variability in both the plasma AUC (x-axis) and biomarker (y-axis) is depicted. Although the model fit of the data shows a very good representation of the mean values in the study, the appropriate use of error bars indicates to the reader that there is variability associated with the predicted response. Based upon these data, a log-linear relationship may have also well described the PD. In this case, additional data points at both higher and lower concentrations might better refine selection of the appropriate model in future studies. Consideration of statistical significance is helpful when interpreting data sets. This statistical analysis will not only aid in the selection of an appropriate model, but also help to guide subsequent study design.

### Translation of preclinical PK/PD to the clinical setting

**Figure 13:**
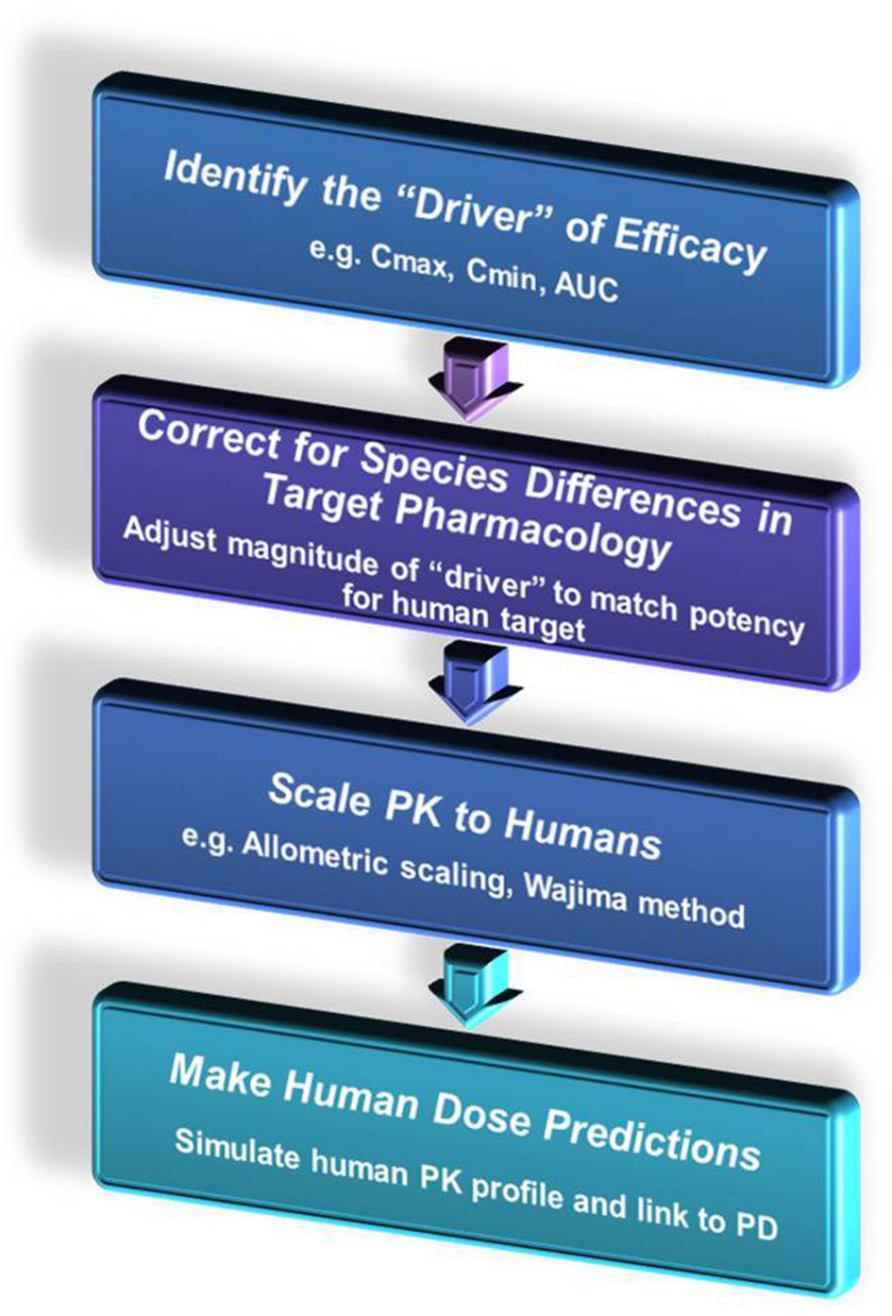
**Extrapolating preclinical PK/PD data to the clinic**.

Once a robust PK/PD relationship has been developed in a preclinical species or relevant model system, these data can be used to help predict anticipated effects in the clinic with some assumptions (Mager and Jusko, 2008; Beaumont and Smith, 2009; Heimbach et al., 2009; Mager et al., 2009; Bueters et al., 2013). Understanding species differences in the biological target, pharmacokinetics, protein binding, and physiology can all aid in more robust translation of preclinical data into the patient population. A general schematic is shown below for extrapolating preclinical PK/PD data to the clinic (Figure 13).

**Figure 14:**
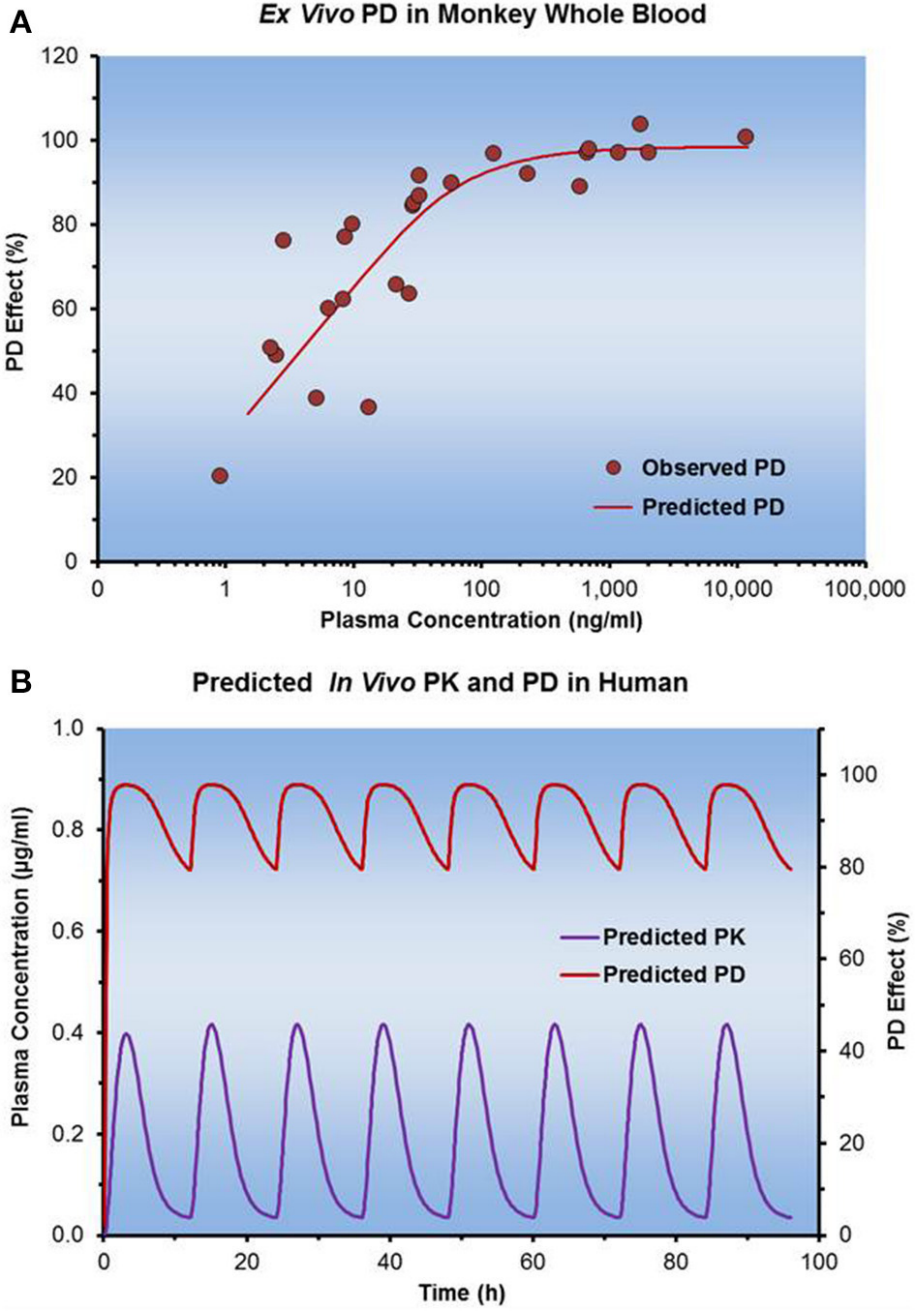
**Translation of preclinical PD data to humans**. **(A)** Illustrates the *ex vivo* incubation data of a drug molecule in whole blood from Cynomolgus monkey when used to determine concentration dependent changes in the desired PD effect. **(B)** Shows how the model fit of PD parameters from the incubations were used to predict the *in vivo* efficacy. Allometric scaling of PK parameters enabled simulations of plasma concentrations in humans.

When human dose predictions were required, a Novartis project team used an approach much like the one illustrated below. The team made the assumptions and showed supporting data that a whole blood PD biomarker could be measured *ex vivo*, that the *ex vivo* response in monkey was translatable to humans, and that the data were best characterized by a direct effect PK/PD model (Figure 14). From the *ex vivo* monkey studies, plasma concentrations needed to be maintained above a C_trough_ value in order to show sustained pharmacological response (>80% inhibition of the target). Assuming that there were no differences in the target pharmacology between monkey and human, corrections for PPB and intrinsic potency across species were made to predict what type of response might be observed *in vivo* in the clinic over a given concentration range. Pharmacokinetics were scaled to humans in using allometric scaling (Mahmood, 2007; Sinha et al., 2008) and the Wajima method (Wajima et al., 2004), thus plasma concentration vs. time profiles were simulated along with anticipated pharmacodynamic response. Equipped with this information, the team could make predictions regarding human dose requirements and anticipated duration of action in the clinic.

## Concluding remarks

Effective translation of preclinical data is critical to the design of appropriate and successful clinical trials. In this review, we emphasize the early implementation of hypothesis-driven preclinical experimental study designs, guided by model-based PK/PD analysis in the drug discovery phase.

A well designed PK/PD strategy enables a project team to design the critical studies needed to address the team's key scientific hypotheses. Incorporating these experimental outcomes back into an established PK/PD framework allows for testing and refinement of the model structure, provides a better mechanistic understanding of the exposure-effect relationship, and most importantly, guides a tailored experimental design to further probe the perturbation of the biological pathway by the compound. This iterative process offers a rational approach to both better understand the mechanism of action of a drug as well as select the optimal compounds for further profiling.

As a drug discovery project moves into the development phase, the sound understanding of the lead compound's PK/PD relationship will provide for a pharmacokinetic basis for anticipating the therapeutic index and aid in pharmacokinetic and biomarker driven design of efficacious dose regimens for clinical proof of concept studies. Several factors are important for maximizing the utility of a rigorous PK/PD analysis. The dataset should adequately capture the following: the entire concentration vs. time and effect vs. time profiles, the model should incorporate a fundamental understanding of the biological pathway being perturbed, a consideration of unbound concentrations, and the statistical and mathematical output should be sound. The ultimate benefit of a PK/PD strategy relies on the continuous integration of experimental outcomes from new compounds in the early discovery phase with the knowledge and results from later stage clinical testing. By incorporating a translational PK/PD framework that can be validated with clinical outcomes, we will improve our ability to treat and cure disease.

### Conflict of interest statement

The authors declare that the research was conducted in the absence of any commercial or financial relationships that could be construed as a potential conflict of interest.
